# P-1802. Assessing the Effectiveness and Essentialness of Antimicrobial Stewardship Practices in Multiple States

**DOI:** 10.1093/ofid/ofae631.1965

**Published:** 2025-01-29

**Authors:** Katie A Thure, Dipen Patel, Lauren Biehle, Christopher D Evans

**Affiliations:** Tennessee Department of Health, Nashville, Tennessee; HAI/AR, Tennessee Department of Health, Nashville, Tennessee; Colorado Department of Public Health and Environment, Denver, Colorado; Tennessee Department of Health, Nashville, Tennessee

## Abstract

**Background:**

Acute Care Hospitals (ACH) self-report on implementation of the Core Elements of Antimicrobial Stewardship through the National Healthcare Safety Network’s (NHSN) Annual Facility Survey. By 2022, a majority of ACHs in Tennessee (TN), Colorado (CO), and Virginia (VA) reported achieving all seven core elements. To gain better insight into their stewardship practices, the Healthcare-Associated Infection Programs from TN, CO, and VA developed a survey assessing the value of specific antimicrobial stewardship practices.

**Table 1.**
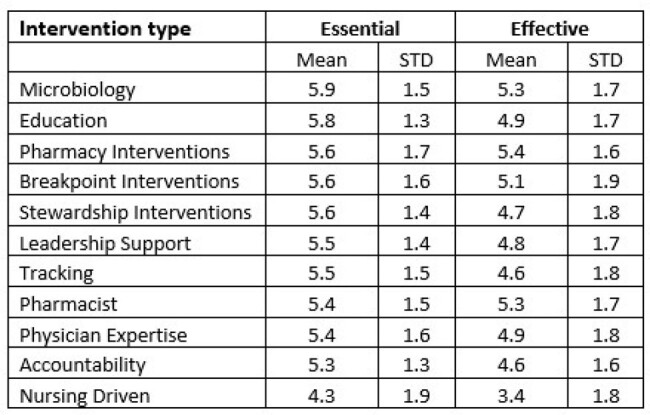
Ratings of how essential and effective interventions are to antibiotic stewardship.

**Methods:**

Key stewards at ACHs were identified and received the survey electronically. Respondents rated how essential each stewardship intervention in the NHSN Survey is to the success of their stewardship program and how effective each specific intervention is at driving antibiotic use at their facility. Respondents rated each intervention from 1 to 7 with 1 being non-essential/effective and 7 being absolutely essential/effective. The overall mean for each category was calculated by averaging the means of each intervention for that specific element.

**Results:**

A total of sixty-four stewards responded to the survey. 82.8% of respondents were Pharmacists. Microbiology based interventions were rated to be the most essential NHSN Survey category with a mean of 5.9, while nurse-driven interventions were the least essential with a mean of 4.3. Pharmacy Interventions were deemed the most effective with a mean of 5.4, whereas Nurse Driven Interventions were also rated the least effective with a mean of 3.4 (Table 1).

**Conclusion:**

Respondents considered all interventions essential. On average, ratings for effectiveness of each intervention were lower than those for essentialness. Microbiology based interventions were rated most essential. This could be attributed to items stewards are able to act on objective data. Even though Nursing Driven interventions had the lowest mean ratings for both essentialness and effectiveness, they also had the lowest number of interventions, which could skew the results. As evidenced by these data, not all stewardship interventions are perceived as equal in driving antimicrobial usage.

**Disclosures:**

**All Authors**: No reported disclosures

